# Prognostic Value of Admission Peak NT-proBNP Combined with Culprit Plaque Types for Predicting Cardiovascular Risk in ST-Segment Elevated Myocardial Infarction: An Optical Coherence Tomography Study

**DOI:** 10.3390/jcdd9120466

**Published:** 2022-12-18

**Authors:** Jiannan Li, Runzhen Chen, Jinying Zhou, Ying Wang, Xiaoxiao Zhao, Chen Liu, Peng Zhou, Yi Chen, Li Song, Shaodi Yan, Hongbing Yan, Hanjun Zhao

**Affiliations:** 1 Department of Cardiology, Fuwai Hospital, National Center for Cardiovascular Diseases, Peking Union Medical College and Chinese Academy of Medical Sciences, Beijing 100037, China; 2 Fuwai Hospital, Chinese Academy of Medical Sciences, Shenzhen 518057, China; 3 Coronary Heart Disease Center, Fuwai Hospital, Chinese Academy of Medical Sciences, Beijing 100037, China

**Keywords:** NT-proBNP, plaque rupture, optical coherence tomography, ST-segment elevated myocardial infarction

## Abstract

Objective: Different culprit plaque phenotypes including plaque rupture (PR) and non-plaque rupture (NPR), and N-Terminal prohormone of brain natriuretic peptide (NT-proBNP) have been reported to influence clinical outcomes in patients with acute coronary syndrome (ACS). We aimed to investigate the prognostic implication of the peak and baseline values at admission for NT-proBNP for major adverse cardiovascular events (MACE) in ST-Segment Elevated Myocardial Infarction (STEMI) patients with different plaque phenotype. Methods: A total of 428 patients with STEMI undergoing optical coherence tomography (OCT) were enrolled and divided into four groups: PR/Tertile1-2 NT-proBNP (*n* = 132), PR/Tertile3 NT-proBNP (*n* = 65), NPR/Tertile1-2 NT-proBNP (*n* = 154), NPR/Tertlie3 NT-proBNP (*n* = 77). Baseline and Peak values of NT-proBNP were obtained in the admission period. The MACEs were defined as the composite of all-cause death, recurrence of myocardial infarction and stroke. Results: High levels for peak NT-proBNP were significantly associated with a higher incidence of MACE and death (Log rank *p* = 0.037 and 0.0012, respectively). In the subgroup with NPR, a high level for peak NT-proBNP was significantly associated with higher incidence of death (Log rank *p* = 0.0022) but this association was not significant in the subgroup of PR (Log rank *p* = 0.24). Though plaque types were not associated with adverse event, the combination of NPR and a higher peak value for NT-proBNP indicated higher incidence of death compared with other groups (Log rank *p* = 0.0017). The area under the receiver operating characteristic curve for predicting death to evaluate the diagnostic value of the peak value for NT-proBNP and plaque types combined with traditional risk factors was 0.843 (95% CI: 0.805–0.876), which is superior to solely traditional risk factors: NRI (26.8% [95% CI: 0.4–53.1%], *p* = 0.046) and IDI (5.1% [95% CI: 1.0–9.2%], *p* = 0.016). Conclusion: STEMI patients with NPR and a high level for peak NT-proBNP showed higher incidence of death. The peak value of NT-proBNP in combination with plaque types can be used in risk stratification and prediction of death in patients with STEMI.

## 1. Introduction

Natriuretic peptides including brain natriuretic peptide (BNP) and N-Terminal prohormone of brain natriuretic peptide (NT-proBNP) have become acknowledged indicators of risk stratification and outcome prediction, not only for heart failure, but also for acute coronary syndrome (ACS) [1,2]. However, in the population of ACS, the prognostic value of NT-proBNP predicting adverse outcome is still controversial [3,4,5,6]. These conflicting results are attributed to the different moment of the NT-proBNP test and the mixture of the study population. The plasma concentration of NT-proBNP changed along with time after onset of myocardial infarction. Most of patients show a unimodal curve with one peak several hours after MI but some patients exhibit a bimodal curve with another peak appearing 3 to 5 days after the first peak [7]. In this study, we continuously recorded the admission value of NT-proBNP every day and selected the baseline and peak value for analysis.

Intracoronary images enable to differentiate the morphology of atherosclerotic plaque and guide precise treatment. Optical coherence tomography (OCT), with the highest resolution, is adept at identifying plaque phenotypes and microstructure in vivo. Moreover, patients with different plaque types had variable clinical outcomes [8]. In the present study, we combined NT-proBNP levels and plaque phenotypes to improve the prediction efficiency of prognosis of patients with acute myocardial infarction (AMI).

## 2. Materials and Methods

### 2.1. Study Population

**Figure 1:**
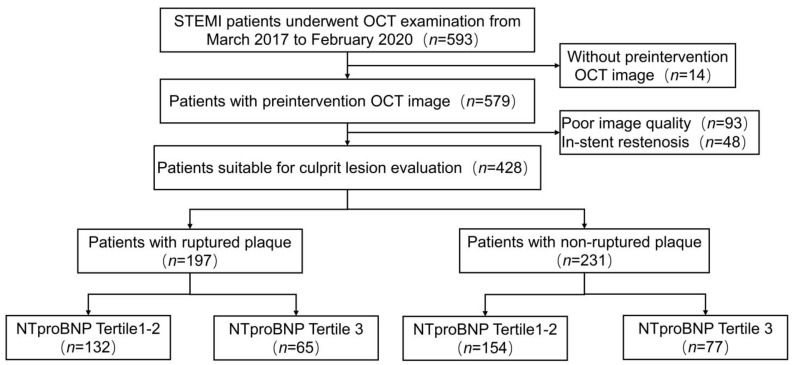
Study flow chart. OCT, optical coherence tomography; STEMI, ST-Segment–Elevation Myocardial Infarction.

From March 2017 to February 2020, 593 ST-Segment Elevated Myocardial Infarction (STEMI) patients with OCT examination were enrolled in our cohort study. A STEMI was defined as continuous chest pain lasting >30 min, ST-segment–elevation >0.1 mV in at least two contiguous leads, or a new left bundle-branch block on the 18-lead ECG, and an elevated troponin I level [9]. We excluded patients with cardiac shock, congestive heart failure, history of coronary artery bypass graft and those with left main diseases, extremely tortuous or heavily calcified vessels, or chronic total occlusion as for our previous study [10]. The study flow chart is displayed in Figure 1.

### 2.2. Acquisition and Analysis of OCT Image

Intravascular OCT imaging was performed in accordance with previously described methods [10]. In brief, following the restoration of antegrade coronary blood flow and reduction in the thrombus burden by pre-dilatation and/or thrombus aspiration, OCT images of culprit lesions were obtained by a frequency-domain OCT system (ILUMIEN OPTIS™; St. Jude Medical/Abbott, St. Paul, MN, USA) and a catheter (Dragonfly™; LightLab Imaging, Inc., Westford, MA, USA). To remove the blood from the field of view and achieve a virtually blood-free environment, continuous flushing with contrast media via manual injection directly through the guiding catheter was conducted during the acquisition of coronary blood vessels while imaging. The images of the entire length of culprit vessels were acquired by using a pullback device that moved at 36 mm/s automatically, and cross-sectional images were generated at a rate of 180 frames/s rotationally. The length of the OCT pullback was 75 mm in total and digitally archived. All OCT images were anonymously analyzed by three independent investigators who were blinded to other clinical data. The first investigator was primarily responsible for screening suitability and location of culprit plaques. The other two investigators analyzed OCT images. Culprit plaques were discriminated based on the coronary angiography and OCT images. The PR was identified as plaque with a disrupted fibrous cap and cavity formation, whereas NPR was defined based on evidence of thrombus, an irregular luminal surface, and no evidence of fibrous cap disruption in multiple adjacent frames [11]. Supplemental Figure S1 shows representative OCT images of PR and NPR.

### 2.3. Laboratory Test

Blood samples were taken by direct venipuncture immediately after admission and every day in the morning in hospital. The samples were collected into EDTA-anticoagulant tubes and the concentration of NT-proBNP was measured by using electrochemiluminescence immunoassay (Elecsys 2010, Roche, Switzerland) according to the manufacturer instructions. Baseline and peak value of NT-proBNP were recorded.

### 2.4. Major Adverse Cardiovascular Events and Follow-Up

Major adverse cardiovascular events (MACEs) were defined as composite all-cause death, recurrence of myocardial infarction, and stroke. Follow-up was performed by well-trained physicians who were blind to clinical data routinely at 1, 6, and 12 months after the discharge via outpatient visits or phone interviews, and then annually, after the 1-year follow-up.

### 2.5. Statistical Analysis

Continuous variables are reported as mean ± standard deviation or median (interquartile range) and categorical variables are presented as numbers (percentages). One-way analysis of variance or Kruskal–Wallis tests were used for comparison of continuous variables. Categorical variables were compared using Pearson chi-square tests or Fisher’s exact test when appropriate. Patients were trisected based on level of baseline and peak value of NT-proBNP in whole population and subgroup of PR and NPR. Tertile 1–2 and Tertile 3 were identified as the low and high NT-proBNP group, respectively. Survival curves were constructed and compared using the Kaplan–Meier method and Log rank test. Univariate and multivariable Cox proportional hazards regression model was used to assess the all-cause death, recurrence of myocardial infarction, stroke, and MACEs composite risk of the four groups, and hazard ratio (HR) and 95% confidence interval (CI) were showed. Subgroup analysis was performed to assess the effect of peak value of NT-proBNP on cardiovascular risk in patients present with different plaque types. Receiver operating characteristic curve (ROC) and area under curve (AUC) were used to evaluate the predictive ability of traditional risk factors and addition of peak value of NT-proBNP combined with plaque types (PR&NPR) for death. Time-dependent receiver operating characteristic (ROC) curves were used to show the predictive power of traditional risk factors and the new model combining traditional risk factors, peak value of NT-proBNP, and plaque types by the method of nearest neighbor estimation. Using net reclassification improvement (NRI) and integrated discrimination improvement (IDI), we also calculated the ability of the new model to reclassify the risk of death in contrast to traditional risk factors. Analyses were conducted using IBM SPSS Statistics version 26.0 (IBM SPSS Statistics, IBM Corporation, Armonk, New York, USA) and R (http://www.r-project.org/, accessed on 10 March 2022) statistical packages. Statistical significance was set at *p*-value of <0.05.

## 3. Results

### 3.1. Baseline Characteristic in Patients Divided by Plaque Types and Levels of NT-proBNP

Of the 579 patients with STEMI who underwent OCT examination before intervention, 141 patients were excluded because of in-stent restenosis (*n* = 48) and poor image quality (*n* = 93). The remaining 428 patients were suitable for plaque morphology evaluation and divided into two groups: patients with ruptured plaque (*n* = 197) and patients with non-ruptured plaque (*n* = 231). Each group was distributed into two subgroups according to the tertile value of the peak NT-proBNP level (Figure 1). Baseline characteristic among these four groups are presented in Table 1 (Group 1: ruptured plaque and tertile 1–2 NT-proBNP [<1872pg/mL]; Group 2: ruptured plaque and tertile 3 NT-proBNP [>1872 pg/mL]; Group 3: non-ruptured plaque and tertile 1–2 NT-proBNP [<723 pg/mL]; Group 4: non-ruptured plaque and tertile 3 NT-proBNP [>723 pg/mL]). Patients in group 4 were more likely to be older and present with higher systemic inflammation. Prevalence of traditional risk factors such as hypertension, diabetes, hyperlipidemia, and smoking showed no difference among these four groups.

### 3.2. Findings with Cox Regression Models in Subgroups

**Figure 2:**
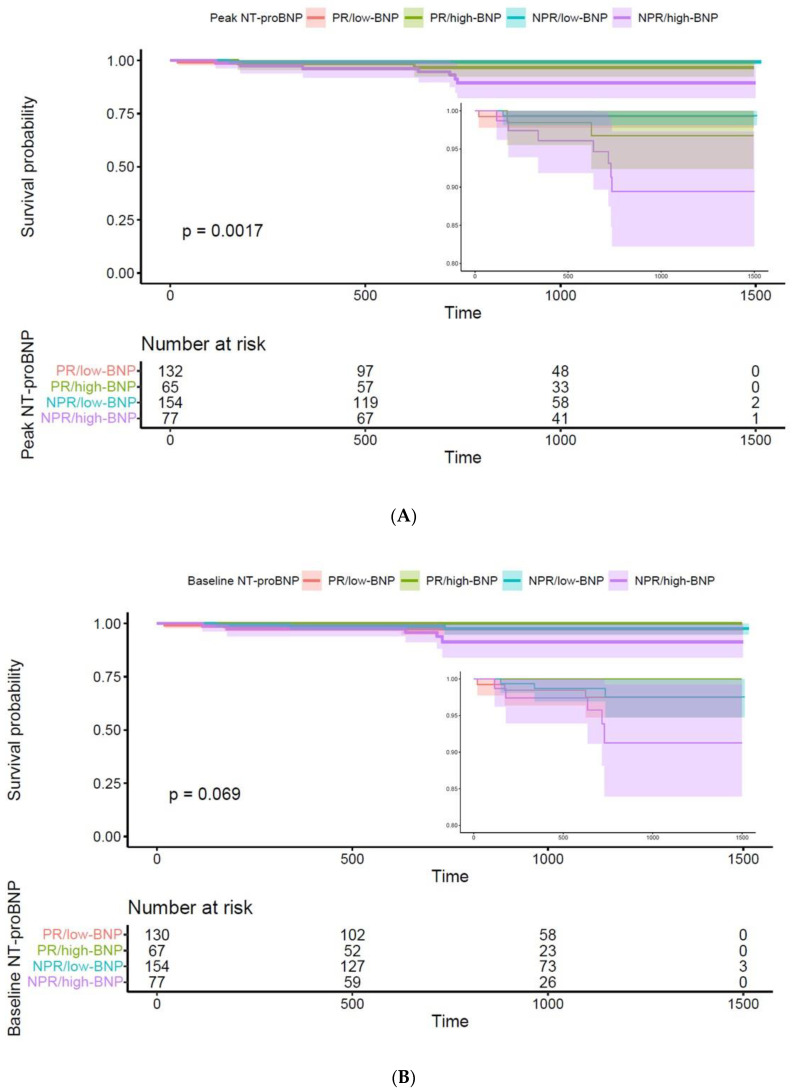
Kaplan–Meier curves for cumulative death combining peak or baseline value of NT-proBNP and plaque types. (**A**) Death for patients of four groups divided by plaque phenotype and peak NT-proBNP; (**B**) Death for patients of four groups divided by plaque phenotype and baseline NT-proBNP. PR, plaque rupture; NPR, non-plaque rupture.

First, we compared the baseline and peak values of NT-proBNP as well as plaque types to predict prognosis of enrolled patients. Our results showed that high levels for peak NT-proBNP were significantly associated with higher incidence of MACEs and death (Log rank *p* = 0.037 and 0.0012, respectively). However, baseline NT-proBNP showed no predictive value to indicate prognosis (Supplemental Figure S2). Next, we added plaque types for further analysis. Alone, plaque types were not associated with adverse events (Log rank *p* = 0.6 and 0.22 for MACEs and death) (Supplemental Figure S3). Besides, baseline NT-proBNP showed no predictive value of death. either in subgroups of PR or NPR. However, in patients with NPR, a high level for peak NT-proBNP was significantly related to incidence of death (Log rank *p* = 0.0022) but this association was not significant in the plaque rupture cohort (Supplemental Figure S4). Finally, all subjects were divided into four groups according to plaque types and the level of peak or baseline NT-proBNP (Figure 2). Patients with NPR and a high level for peak NT-proBNP showed a significantly higher incidence of death comparing with other groups (Log rank *p* = 0.0017).

Then, subjects divided by the level of the peak value of NT-proBNP were compared in the subgroups of PR and NPR. In patients with PR, there was no significant difference of MACEs and its composition between low and high level of NT-proBNP. However, in patients with NPR, a higher level of NT-proBNP showed increasing risk of death and MACE composite than a lower level of NT-proBNP (Supplemental Table S1). Moreover, the crude and adjusted models of MACE according to plaque types and peak value of NT-proBNP were established and are exhibited in Table 2. The NPR patients with a high peak value of NT-proBNP were associated with a higher cumulative incidence of death over time (HR = 10.971, 95% CI: 1.348–89.262, *p* = 0.025 in crude model; HR = 11.298; 95% CI: 1.155–110.546, *p* = 0.037 after full adjustment). However, there was no significance of incidence of myocardial infarction, stroke, and MACE composite among the four groups.

### 3.3. Diagnostic Value of Level of NT-proBNP in Combination with Plaque Types

**Figure 3:**
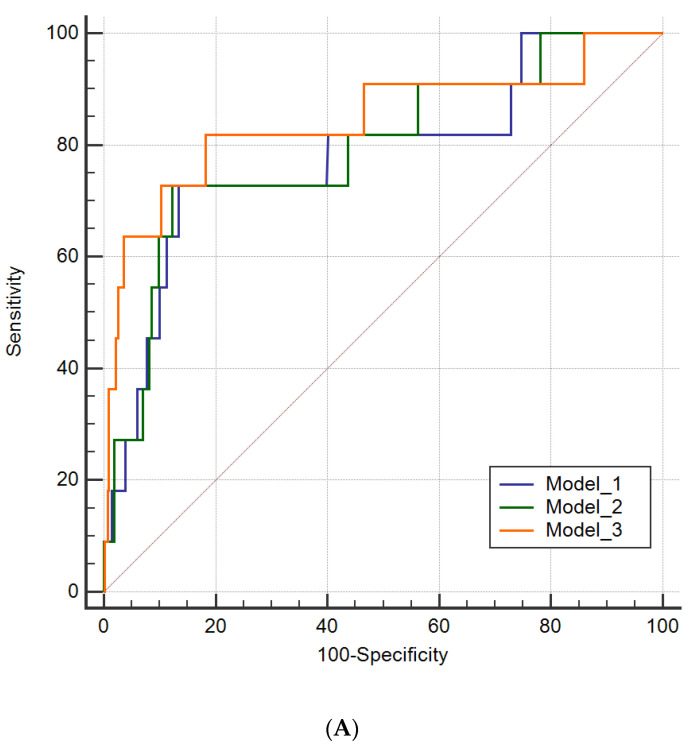

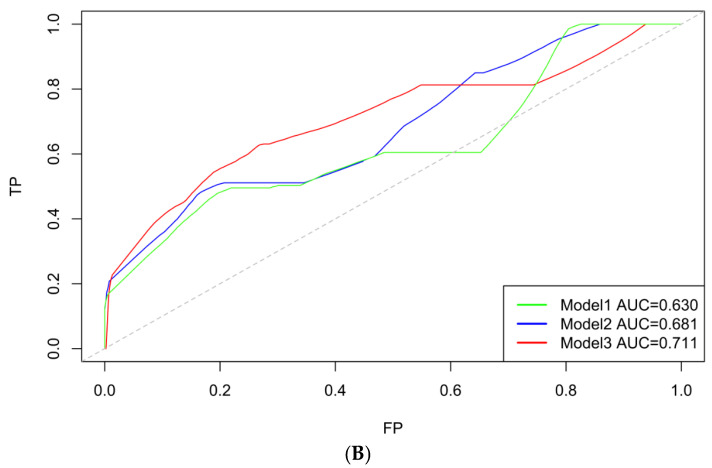
ROC curve (**A**) and time-dependent ROC curves (**B**) by different models for adverse clinical outcome. (Model 1, predictor of traditional risk factors including age, gender, hypertension, hyperlipidemia, diabetes, smoke, LDL, Hs-CRP, LVEF; Model 2, model 1 plus NT-proBNP; Model 3, model 2 plus plaque).

The ROC curves were plotted to evaluate the diagnostic value of traditional risk factors and in combination with the peak value of NT-proBNP and plaque types (Figure 3A). The AUC of Model 1 containing risk factors (including age, gender, hypertension, hyperlipidemia, diabetes, smoke, LDL, Hs-CRP, LVEF) was 0.780 (95% CI: 0.738–0.819). Model 2 was established by adding the peak value of NT-proBNP and its AUC was 0.793 (95% CI: 0.751–0.830). While adding plaque types to Model 2, the AUC of Model 3 increased to 0.843 (95% CI: 0.805–0.876). The addition of the peak value of NT-proBNP and plaque types to the traditional risk factors model also resulted in a significant increase in the NRI (26.8% [95% CI: 0.4–53.1%], *p* = 0.046) and IDI (5.1% [95% CI: 1.0–9.2%], *p* = 0.016) (Supplemental Table S2). Furthermore, time-dependent ROC curves of traditional risk factors and addition of the peak value of NT-proBNP and plaque types for 3-year death occurrences are shown in Figure 3B. Traditional risk factors plus the peak value of NT-proBNP and plaque types presented higher AUC to predict death than the other two models (AUC of Model1 vs. Model2 vs. Model3 = 0.630 vs. 0.681 vs. 0.711) but showed no significant difference between every combination of two models (p _Model1 vs. Model2_ = 0.555, p _Model2 vs. Model3_ = 0.387, p _Model1 vs. Model3_ = 0.398).

Group 1 indicated plaque rupture and Tertile 1–2 NT-proBNP; Group 2 indicated plaque rupture and Tertile3 NT-proBNP; Group 3 indicated non plaque rupture and Tertile 1–2 NT-proBNP; Group 4 indicated non plaque rupture and Tertlie3 NT-proBNP. Continuous data are presented as median (interquartile range). Categorical data are presented as number (%), BMI body mass index, PCI percutaneous coronary intervention, CKD chronic kidney disease, WBC white blood cell, HDL, high density lipoprotein, LDL low density lipoprotein, hs-CRP high sensitive C-reactive protein, ACEI angiotensin-converting enzyme inhibitor, ARB angiotensin receptor blocker, HbA1c Hemoglobin A1c, PPI proton pump inhibitors, LAD left anterior descending artery, LCX left circumfex artery, RCA right coronary artery, TIMI thrombolysis in myocardial infarction * *p* < 0.05.

## 4. Discussion

The NT-proBNP contains 76 amino acid and is produced by degradation of proBNP, which is more stable, less impacted by other proteins in circulation and had longer half-time than BNP. The use of NT-proBNP has become a powerful diagnostic and prognostic indicator of heart failure in the past few decades [12,13]. Moreover, the level of circulating NT-proBNP has been found to be associated with short- and long-term prognosis of patients with AMI due to its particularity of reflecting cardiac impairment and remodeling [14,15]. The concentration of BNP and NT-proBNP increase after the onset of myocardial infarction probably due to cardiac injury, secretion of cytokines and remodeling of ventricle [16,17]. Most AMI patients have one or two peaks of BNP after onset of myocardial infarction. The first peak is associated with myocardial injury in the acute phase and the second peak, appearing several days after the first peak, is related to myocardial remodeling which is almost seen in anterior myocardial infarction and reflects the degree of left ventricular dysfunction or the size of the infarction [7,18].

The considerable variability of NT-proBNP values among individuals has made it difficult to select an optimum threshold for precise prediction of short- and long-term outcomes [19]. In the past few decades, many studies have explored the different timepoints of admission or follow-up NT-proBNP for identification of high-risk patients. Values of NT-proBNP at baseline, 24 h, and 72h after onset of STEMI are reported to be independently associated with short-term outcome and cardiac function [19]. However, in our study, baseline NT-proBNP exhibited a weak association with clinical outcome. Another previous study, with a small sample size, demonstrated that later measurement of NT-proBNP, after AMI (73–120 h), better predicted and adverse outcome than an immediate measurement [20]. Furthermore, Wojciech Drewniak found that the level of NT-proBNP within 1 day after myocardial infarction was significantly associated with the long-term outcome [21]. Moreover, the plasma level of BNP obtained 3 to 4 weeks after the onset of AMI can also precisely predict cardiac death in patients with AMI [18]. Omland et al. found that NT-proBNP obtained in the subacute phase (median 3 days) of ACS can also predict long-term prognosis of patients [22]. However, whether the peak value of admission NT-proBNP after the onset of AMI can be a reliable prognostic indicator remains unclear. In this study, we consistently recorded the admission NT-proBNP of AMI patients and found that a high level of the peak NT-proBNP value was significantly related to long-term death and MACE. Additionally, almost all the previous studies did not triage acute myocardial infarction based on morphological features of culprit lesions, which might lead to some controversial results [3,23]. While taking plaque types into consideration, a high level of the peak value of NT-proBNP was significantly related to worse prognosis in the population with NPR, but in patients with PR, NT-proBNP showed weak predictive value for MACE.

The PR accounts for approximately two-thirds of acute coronary syndrome and other etiologies involve in plaque erosion, calcified nodules, coronary spasm, and microcirculation dysfunction. Discrimination of plaque types and characteristics enables to improve the risk stratification and precise management of patients with ACS. Niccoli et al. reported that plaque rupture portended worse outcomes than intact fibrous cap in patients with acute coronary syndrome [8]. Besides, in population with an intact fibrous cap, presence of lipid-rich plaque had worse outcome than those without lipid-rich plaque [24]. Furthermore, patients with plaque erosion were associated with less microvascular damage after PCI and remained free of adverse cardiac events for up to 1 year [25,26]. However, a retrospective study of enrolled patients with ACS showed no difference of long-term outcome between plaque rupture and erosion [25], which was consistent with our results. Besides, another study drew a similar conclusion and patients with a calcified nodule were associated with more adverse events than plaque rupture and erosion [27]. This discrepancy might mainly be due to the different sample size and follow-up time as well as population race. Although patients with different plaque types in our study did not show a significant distinction of long-term MACE and death, however, while combining with a high value for peak NT-proBNP, the NPR could indicate higher incidence of death than other groups. This reflected that plaque types and NT-proBNP co-impacted the prognosis of patients with STEMI. Previous study has indicated that patients with plaque rupture presented larger transmural extent of infarction than those without plaque rupture [28]. Besides, vulnerable culprit lesion morphology was a consistent determinant of advanced myocardial infarction and injury [29]. These results indicated that plaque rupture usually accompanied with worse cardiac function. However, there has been no study indicating the significance of cardiac function in prognosis of patients with PR and NPR. Our results, for the first time, show that patients with NPR and higher NT-proBNP had the worst clinical outcome. Therefore, assessing cardiovascular risk from both plaque phenotype and the level of NT-proBNP is a more comprehensive risk stratification strategy. However, the mechanism needs further experimental and clinical investigation.

The present study not only evaluated the prognostic value of peak NT-proBNP levels in combination with plaque types on OCT, but also provided a novel method for the risk stratification of patients with STEMI who are undergoing primary PCI. Adding culprit plaque types by OCT to the peak value of NT-proBNP, together with the traditional cardiovascular risk factors, could improve the predictive power for clinical outcomes.

This study has several potential limitations. First, the it was a single-center study, and nearly one-quarter of patients were excluded from the final analysis. Therefore, selection bias could not be avoided. Second, thrombus aspiration and balloon dilation before OCT in some cases may modify the plaque, which might lead to misclassification. Third, the underlying plaque morphology of the culprit lesion could have been covered by residual thrombus in some cases. Therefore, some ruptured plaque may be misjudged as non-ruptured plaque. Fourth, an independent cohort to validate the value of plaque types and the peak value of NT-proBNP to predict clinical outcome is missing, which we aim to do in future studies.

## 5. Conclusions

The present study strongly suggested the peak value of NT-proBNP is predictive of MACEs, especially death in patients with STEMI and the subgroup of NPR. Patients with NPR and a high level of peak value of NT-proBNP showed significantly higher incidence of death. The model of traditional risk factors plus plaque types and the peak value of NT-proBNP showed excellent performance for predicting long-term mortality of STEMI patients.

## Figures and Tables

**Table 1:** Baseline clinical characteristics of the study population.

Variable	Total (*n* = 428)	Group 1 (*n* = 132)	Group 2 (*n* = 65)	Group 3 (*n* = 154)	Group 4 (*n* = 77)	*p* Value
Demographic data						
Age (years)	58.1 ± 11.9	55.9 ± 12.2	61.0 ± 12.1	56.5 ± 11.4	62.4 ± 10.8	<0.001
Male [%(*n*)]	353 (82.5)	112 (84.8)	52 (80.0)	131 (85.1)	58 (75.3)	0.238
BMI (kg/m^2^)	26.0 ± 3.3	26.5 ± 3.3	25.0 ± 3.2	26.2 ± 3.3	25.4 ± 3.3	0.922
Risk factors						
Hypertension [%(*n*)]	253 (59.1)	73 (55.3)	43 (66.2)	84 (54.5)	53 (68.8)	0.091
Diabetes [%(*n*)]	127 (29.7)	42 (31.8)	16 (24.6)	46 (29.9)	23 (29.9)	0.779
Hyperlipidemia [%(*n*)]	382 (89.3)	122 (92.4)	56 (86.2)	135 (87.7)	69 (89.6)	0.484
Smoking [%(*n*)]	311 (72.8)	97 (73.5)	48 (73.8)	114 (74.5)	52 (67.5)	0.712
Previous PCI [%(*n*)]	40 (9.3)	13 (9.8)	7 (10.8)	12 (7.8)	8 (10.4)	0.866
CKD [%(*n*)]	10 (2.3)	5 (3.8)	3 (4.6)	1 (0.6)	1 (1.3)	0.175
Stroke [%(*n*)]	37 (8.7)	8 (6.1)	5 (7.7)	15 (9.7)	9 (11.8)	0.493
Laboratory Findings						
HDL-C (mmol/L)	1.1 ± 0.3	1.1 ± 0.2	1.1 ± 0.2	1.1 ± 0.4	1.1 ± 0.4	0.571
LDL-C (mmol/L)	2.8 ± 0.8	2.9 ± 0.8	2.8 ± 0.9	2.7 ± 0.8	2.5 ± 0.9	0.032
Triglycerides (mmol/L)	1.7 ± 1.2	1.9 ± 1.2	1.6 ± 1.1	1.7 ± 1.2	1.4 ± 0.9	0.011
hs-CRP (mg/L)	5.9 (2.3, 10.9)	4.2 (2.3, 9.6)	7.2 (1.6, 10.9)	5.4 (1.8, 10.6)	10.0 (3.7, 12.3)	<0.001
WBC 10^9^/L	9.8 ± 3.0	9.5 ± 2.9	10.4 ± 3.0	9.8 ± 3.0	9.8 ± 3.0	0.909
Hemoglobin	143.4 ± 17.2	145.5 ± 16.5	137.9 ± 21.7	144.7 ± 15.7	141.8 ± 16.3	0.017
Platelet	233.2 ± 63.6	236.4 ± 61.6	215.9 ± 56.9	241.9 ± 67.0	224.7 ± 62.8	0.024
Creatine (umol/L)	81.2 (69.7, 92.5)	80.4 (71.2, 91.4)	84.4 (70.0, 98.5)	78.8 (68.6, 89.0)	85.7 (69.8, 98.6)	0.083
Glucose (mmol/L)	8.5 ± 3.6	8.3 ± 3.4	9.0 ± 4.5	8.4 ± 3.3	8.6 ± 3.4	0.659
HAb1c (%)	6.6 ± 1.5	6.7 ± 1.7	6.4 ± 1.2	6.7 ± 1.5	6.4 ± 1.6	0.397
Discharge medication						
Aspirin	414 (96.7)	129 (97.7)	63 (96.9)	147 (95.5)	75 (97.4)	0.723
Ticagrelor [%(*n*)]	219 (51.2)	73 (55.3)	37 (56.9)	71 (46.1)	38 (49.4)	0.328
Clopidogrel [%(*n*)]	207 (48.4)	58 (43.9)	28 (43.1)	82 (53.2)	39 (50.6)	0.335
ACEI/ARB [%(*n*)]	320 (74.8)	103 (78.0)	46 (70.8)	115 (74.7)	56 (72.7)	0.690
Beta-Blockers [%(*n*)]	371 (86.7)	114 (86.4)	60 (92.3)	133 (86.4)	64 (83.1)	0.448
Statin [%(*n*)]	414 (96.7)	127 (96.2)	64 (98.5)	148 (96.1)	75 (97.4)	0.794
PPI [%(*n*)]	203 (47.4)	55 (41.7)	37 (56.9)	71 (46.1)	40 (51.9)	0.183
Culprit vessels						0.009
LAD	198 (46.7)	44 (33.8)	38 (58.5)	72 (47.4)	44 (57.1)	
LCX	49 (11.6)	17 (13.1)	9 (13.8)	19 (12.5)	4 (5.2)	
RCA	170 (40.1)	66 (50.8)	18 (27.7)	57 (37.5)	29 (37.7)	
Syntax score	16.0 (11.0, 22.5)	16.0 (11.0, 23.5)	17.5 (11.0, 22.5)	15.8 (10.4, 21.8)	15.0 (10.0, 22.5)	0.780
Pre TIMI flow						0.726
0	256 (61.8)	70 (55.1)	44 (67.7)	89 (61.0)	53 (69.7)	
1	19 (4.6)	6 (4.7)	2 (3.1)	8 (5.5)	3 (3.9)	
2	45 (10.9)	16 (12.6)	7 (10.8)	16 (11.0)	6 (7.9)	
3	94 (22.7)	35 (27.6)	12 (18.5)	33 (22.6)	14 (18.4)	
Diameter of lesion	2.7 ± 1.1	2.6 ± 1.2	2.8 ± 0.8	2.7 ± 1.2	3.0 ± 0.8	0.165
Length of lesion	28.9 ± 14.5	26.3 ± 13.0	29.6 ± 16.7	29.2 ± 13.9	32.0 ± 15.6	0.053

**Table 2:** Association between separate endpoints survival and groups which divided by plaque types and peak NT-proBNP in all enrolled patients.

Group	Crude Model		Adjust Model I		Adjust Model II		Adjust Model III	
	Crude HR (95%CI)	Crude *p* Value	Adjust I HR (95%CI)	Adjust *p* Value	Adjust II HR (95%CI)	Adjust *p* Value	Adjust III HR (95%CI)	Adjust *p* Value
Death								
1	1 (reference)	-	1 (reference)	-	1 (reference)	-	1 (reference)	-
2	3.790 (0.343–41.810)	0.277	2.2897 (0.260–32.237)	0.387	2.650 (0.234–29.978)	0.431	2.469 (0.204–29.840)	0.477
3	0.851 (0.053–13.612)	0.909	0.829 (0.052–13.260)	0.895	0.888 (0.055–14.319)	0.934	0.976 (0.059–16.173)	0.986
4	10.971 (1.348–89.262)	0.025 *	7.804 (0.938–64.950)	0.057	9.132 (1.101–75.758)	0.040*	11.298 (1.155–110.546)	0.037 *
Trend test	2.271 (1.138–4.535)	0.020 *	1.979 (1.015–3.857)	0.045 *	2.156 (1.093–4.254)	0.027*	2.278 (1.065–4.869)	0.034 *
MI								
1	1 (reference)	-	1 (reference)	-	1 (reference)	-	1 (reference)	-
2	1.461 (0.327–6.531)	0.620	1.165 (0.256–5.301)	0.844	1.157 (0.232–5.786)	0.859	1.453 (0.236–8.941)	0.687
3	0.846 (0.212–3.382)	0..813	0.815 (0.203–3.262)	0.772	0.730 (0.175–3.041)	0.665	0.838 (0.169–4.149)	0.828
4	1.237 (0.277–5.532)	0.781	0.940 (0.204–4.323)	0.936	1.213 (0.252–5.841)	0.810	0.934 (0.120–7.281)	0.948
Trend test	1.005 (0.625–1.616)	0.984	0.944 (0.587–1.518)	0.811	0.983 (0.597–1.620)	0.947	0.906 (0.507–1.618)	0.739
Stroke								
1	1 (reference)	-	1 (reference)	-	1 (reference)	-	1 (reference)	-
2	0.579 (0.060–5.575)	0.637	0.499 (0.051–4.869)	0.550	0.521 (0.052–5.201)	0.578	0.430 (0.036–5.152)	0.505
3	0.839 (0.169–4.156)	0.829	0.823 (0.166–4.077)	0.811	0.871 (0.173–4.384)	0.867	0.764 (0.146–4.001)	0.750
4	1.971 (0.440–8.823)	0.375	1.565 (0.336–7.294)	0.568	1.451 (0.304–6.936)	0.641	1.460 (0.275–7.756)	0.657
Trend test	1.263 (0.727–2.195)	0.407	1.189 (0.686–2.061)	0.538	1.166 (0.677–2.010)	0.579	1.179 (0.667–2.084)	0.570
MACE composite								
1	1 (reference)	-	1 (reference)	-	1 (reference)	-	1 (reference)	-
2	1.364 (0.473–3.933)	0.566	1.095 (0.375–3.198)	0.869	1.147 (0.386–3.410)	0.805	1.093 (0.344–3.474)	0.881
3	0.836 (0.314–2.229)	0.721	0.837 (0.314–2.231)	0.722	0.837 (0.313–2.239)	0.723	0.927 (0.333–2.581)	0.885
4	2.177 (0.900–5.264)	0.084	1.698 (0.685–4.207)	0.253	1.881 (0.749–4.727)	0.179	1.713 (0.591–4.966)	0.322
Trend test	1.237 (0.910–1.680)	0.175	1.160 (0.855–1.573)	0.342	1.187 (0.871–1.618)	0.278	1.153 (0.824–1.614)	0.406

Data presented are HRs and 95% CI. Adjust I model adjusts for sex and age; Adjust II model adjusts for adjust I plus smoke, hypertension, hyperlipidemia, diabetes mellitus and BMI; Adjust III model adjusts for adjust II + white blood cell counts, creatine kinase, glycosylated hemoglobin, high sensitivity C-reactive protein and left ventricle ejection fraction. Group = 1 (PR and low NT-proBNP), Group = 2 (PR and high NT-proBNP), Group = 3 (NPR and low NT-proBNP), Group = 4 (NPR and high NT-proBNP) * *p* < 0.05.

## Data Availability

The data used to support the findings of this study are available from the corresponding authors upon request. The institution (Fuwai Hospital) requires all requests for accessing any data of patients to be applied for and processed in a case-by-case manner.

## Supplementary Materials

The following supporting information can be downloaded at https://www.mdpi.com/article/10.3390/jcdd9120466/s1. Figure S1. Representative optical coherence tomography images for plaque rupture and non-plaque rupture. (A–C) Plaque rupture; (D–F) Non-plaque rupture. Figure S2. Kaplan–Meier curves for cumulative rates of MACE and death according to peak and baseline value of NT-proBNP. (A) MACE for peak value of NT-proBNP; (B) Death for peak value of NT-proBNP; (C) Death for baseline value of NT-proBNP; (D) MACE for baseline value of NT-proBNP. PNT-proBNP, peak value of NT-proBNP; BNT-proBNP, baseline value of NT-proBNP. Figure S3. Kaplan–Meier curves for cumulative rates of MACE and death according to plaque phenotype. (A) MACE for plaque rupture; (B) Death for non-plaque rupture. PR, plaque rupture; NPR, non-plaque rupture. Figure S4. Kaplan–Meier curves for cumulative death rate according to peak or baseline value of NT-proBNP and plaque phenotype. (A) Death for baseline value of NT-proBNP in patients with PR; (B) Death for baseline value of NT-proBNP in patients with NPR; (C) Death for peak value of NT-proBNP in patients with PR; (D) Death for peak value of NT-proBNP in patients with NPR. PR, plaque rupture; NPR, non-plaque rupture. Table S1. Hazard Ratio to MACE According to peak value of NT-proBNP in Plaque Rupture and Non-Plaque rupture. Table S2. Discrimination and Reclassification of 2-Year death by Different Models.

## References

[1] Maisel A.S., Krishnaswamy P., Nowak R.M., McCord J., Hollander J.E., Duc P., Omland T., Storrow A.B., Abraham W.T., Wu A.H. (2002). Rapid Measurement of B-Type Natriuretic Peptide in the Emergency Diagnosis of Heart Failure. N. Engl. J. Med..

[2] Richards A.M., Nicholls M.G., Yandle T.G., Frampton C., Espiner E.A., Turner J.G., Buttimore R.C., Lainchbury J.G., Elliott J.M., Ikram H. (1998). Plasma N-Terminal Pro–Brain Natriuretic Peptide and Adrenomedullin: New neurohormonal predictors of left ventricular function and prognosis after myocardial infarction. Circulation.

[3] Latini R., Maggioni A.P., Peri G., Gonzini L., Lucci D., Mocarelli P., Vago L., Pasqualini F., Signorini S., Soldateschi D. (2004). Prognostic Significance of the Long Pentraxin PTX3 in Acute Myocardial Infarction. Circulation.

[4] Brügger-Andersen T., Aarsetøy H., Grundt H., Staines H., Nilsen D.W. (2008). The long-term prognostic value of multiple biomarkers following a myocardial infarction. Thromb. Res..

[5] Collet J.-P., Thiele H., Barbato E., Barthélémy O., Bauersachs J., Bhatt D.L., Dendale P., Dorobantu M., Edvardsen T., Folliguet T. (2021). 2020 ESC Guidelines for the management of acute coronary syndromes in patients presenting without persistent ST-segment elevation. Eur. Heart J..

[6] Khan S.Q., Kelly D., Quinn P., Davies J.E., Ng L. (2007). Myotrophin is a more powerful predictor of major adverse cardiac events following acute coronary syndrome than N-terminal pro-B-type natriuretic peptide. Clin. Sci..

[7] Morita E., Yasue H., Yoshimura M., Ogawa H., Jougasaki M., Matsumura T., Mukoyama M., Nakao K. (1993). Increased plasma levels of brain natriuretic peptide in patients with acute myocardial infarction. Circulation.

[8] Niccoli G., Montone R.A., Di Vito L., Gramegna M., Refaat H., Scalone G., Leone A.M., Trani C., Burzotta F., Porto I. (2015). Plaque rupture and intact fibrous cap assessed by optical coherence tomography portend different outcomes in patients with acute coronary syndrome. Eur. Hear. J..

[9] Ibanez B., James S., Agewall S., Antunes M.J., Bucciarelli-Ducci C., Bueno H., Caforio A.L.P., Crea F., Goudevenos J.A., Halvorsen S. (2018). 2017 ESC Guidelines for the management of acute myocardial infarction in patients presenting with ST-segment elevation: The Task Force for the management of acute myocardial infarction in patients presenting with ST-segment elevation of the European Society of Cardiology (ESC). Eur. Heart J..

[10] Tan Y., Sheng Z., Zhou P., Liu C., Zhao H., Song L., Li J., Zhou J., Chen Y., Wang L. (2019). Plasma Trimethylamine N-Oxide as a Novel Biomarker for Plaque Rupture in Patients With ST-Segment–Elevation Myocardial Infarction. Circ. Cardiovasc. Interv..

[11] Tearney G.J., Regar E., Akasaka T., Adriaenssens T., Barlis P., Bezerra H.G., Bouma B., Bruining N., Cho J.-M., Chowdhary S. (2012). Consensus Standards for Acquisition, Measurement, and Reporting of Intravascular Optical Coherence Tomography Studies: A Report from the International Working Group for Intravascular Optical Coherence Tomography Standardization and Validation. J. Am. Coll. Cardiol..

[12] McDonagh T.A., Metra M., Adamo M., Gardner R.S., Baumbach A., Böhm M., Burri H., Butler J., Čelutkienė J., Chioncel O. (2021). 2021 ESC Guidelines for the diagnosis and treatment of acute and chronic heart failure. Eur. Heart J..

[13] Yancy C.W., Jessup M., Bozkurt B., Butler J., Casey D.E., Drazner M.H., Fonarow G.C., Geraci S.A., Horwich T., Januzzi J.L. (2013). 2013 ACCF/AHA guideline for the management of heart failure: A report of the American College of Cardiology Foundation/American Heart Association Task Force on Practice Guidelines. J. Am. Coll. Cardiol..

[14] Hunt P.J., Richards A.M., Nicholls M.G., Yandle T.G., Doughty R.N., Espiner E.A. (1997). Immunoreactive amino-terminal pro-brain natriuretic peptide (NT-PROBNP): A new marker of cardiac impairment. Clin. Endocrinol..

[15] Parenica J., Kala P., Pavkova M.G., Tomandl J., Spinar J., Littnerova S., Jarkovsky J., Mebazaa A., Tomandlova M., Dastych M. (2016). Natriuretic peptides, nitrite/nitrate and superoxide dismutase have additional value on top of the GRACE score in prediction of one-year mortality and rehospitalisation for heart failure in STEMI patients—Multiple biomarkers prospective cohort study. Int. J. Cardiol..

[16] Nakagawa O., Ogawa Y., Itoh H., Suga S., Komatsu Y., Kishimoto I., Nishino K., Yoshimasa T., Nakao K. (1995). Rapid transcriptional activation and early mRNA turnover of brain natriuretic peptide in cardiocyte hypertrophy. Evidence for brain natriuretic peptide as an "emergency" cardiac hormone against ventricular overload. J. Clin. Investig..

[17] Harada E., Nakagawa O., Yoshimura M., Harada M., Nakagawa M., Mizuno Y., Shimasaki Y., Nakayama M., Yasue H., Kuwahara K. (1999). Effect of Interleukin-1β on Cardiac Hypertrophy and Production of Natriuretic Peptides in Rat Cardiocyte Culture. J. Mol. Cell. Cardiol..

[18] Suzuki S., Yoshimura M., Nakayama M., Mizuno Y., Harada E., Ito T., Nakamura S., Abe K., Yamamuro M., Sakamoto T. (2004). Plasma Level of B-Type Natriuretic Peptide as a Prognostic Marker after Acute Myocardial Infarction: A long-term follow-up analysis. Circulation.

[19] Ezekowitz J.A., Théroux P., Chang W., Mahaffey K.W., Granger C.B., Weaver W., Hochman J.S., Armstrong P.W. (2006). N-terminal pro-brain natriuretic peptide and the timing, extent and mortality in ST elevation myocardial infarction. Can. J. Cardiol..

[20] Talwar S., Squire I., Downie P., McCullough A., Campton M., Davies J., Barnett D., Ng L. (2000). Profile of plasma N-terminal proBNP following acute myocardial infarction. Correlation with left ventricular systolic dysfunction. Eur. Hear. J..

[21] Drewniak W., Szybka W., Bielecki D., Malinowski M., Kotlarska J., Krol-Jaskulska A., Popielarz-Grygalewicz A., Konwicka A., Dąbrowski M. (2015). Prognostic Significance of NT-proBNP Levels in Patients over 65 Presenting Acute Myocardial Infarction Treated Invasively or Conservatively. BioMed Res. Int..

[22] Omland T., Persson A., Ng L., O’Brien R., Karlsson T., Herlitz J., Hartford M., Caidahl K. (2002). N-Terminal Pro-B–Type Natriuretic Peptide and Long-Term Mortality in Acute Coronary Syndromes. Circulation.

[23] Shen S., Ye J., Wu X., Li X. (2021). Association of N-terminal pro-brain natriuretic peptide level with adverse outcomes in patients with acute myocardial infarction: A meta-analysis. Hear. Lung.

[24] Hoshino M., Yonetsu T., Usui E., Kanaji Y., Ohya H., Sumino Y., Yamaguchi M., Hada M., Hamaya R., Kanno Y. (2019). Clinical Significance of the Presence or Absence of Lipid-Rich Plaque Underneath Intact Fibrous Cap Plaque in Acute Coronary Syndrome. J. Am. Hear. Assoc..

[25] Hu S., Zhu Y., Zhang Y., Dai J., Li L., Dauerman H., Soeda T., Wang Z., Lee H., Wang C. (2017). Management and Outcome of Patients with Acute Coronary Syndrome Caused by Plaque Rupture Versus Plaque Erosion: An Intravascular Optical Coherence Tomography Study. J. Am. Hear. Assoc..

[26] Higuma T., Soeda T., Abe N., Yamada M., Yokoyama H., Shibutani S., Vergallo R., Minami Y., Ong D.S., Lee H. (2015). A Combined Optical Coherence Tomography and Intravascular Ultrasound Study on Plaque Rupture, Plaque Erosion, and Calcified Nodule in Patients with ST-Segment Elevation Myocardial Infarction: Incidence, Morphologic Characteristics, and Outcomes after Percutaneous Coronary Intervention. JACC: Cardiovasc. Interv..

[27] Nagasawa A., Otake H., Kawamori H., Toba T., Sugizaki Y., Takeshige R., Nakano S., Tanimura K., Takahashi Y., Fukuyama Y. (2021). Relationship among clinical characteristics, morphological culprit plaque features, and long-term prognosis in patients with acute coronary syndrome. Int. J. Cardiovasc. Imaging.

[28] Satogami K., Ino Y., Kubo T., Tanimoto T., Orii M., Matsuo Y., Ota S., Yamaguchi T., Shiono Y., Shimamura K. (2017). Impact of Plaque Rupture Detected by Optical Coherence Tomography on Transmural Extent of Infarction after Successful Stenting in ST-Segment Elevation Acute Myocardial Infarction. JACC Cardiovasc. Interv..

[29] Okada K., Hibi K., Kikuchi S., Kirigaya H., Hanajima Y., Sato R., Nakahashi H., Minamimoto Y., Matsuzawa Y., Maejima N. (2022). Culprit Lesion Morphology of Rapidly Progressive and Extensive Anterior-Wall ST-Segment Elevation Myocardial Infarction. Circ. Cardiovasc. Imaging.

