# Supplementary material for: Prognostic Value of Admission Peak NT-proBNP Combined with Culprit Plaque Types for Predicting Cardiovascular Risk in ST-Segment Elevated Myocardial Infarction: An Optical Coherence Tomography Study
Source: J Cardiovasc Dev Dis. 2022 Dec 18;9(12):466. doi: 10.3390/jcdd9120466 (PMC9786275; doi:10.3390/jcdd9120466)
Supplement: Supplementary file 1 [file jcdd-09-00466-s001.zip › jcdd-2041908-supplementary.pdf]

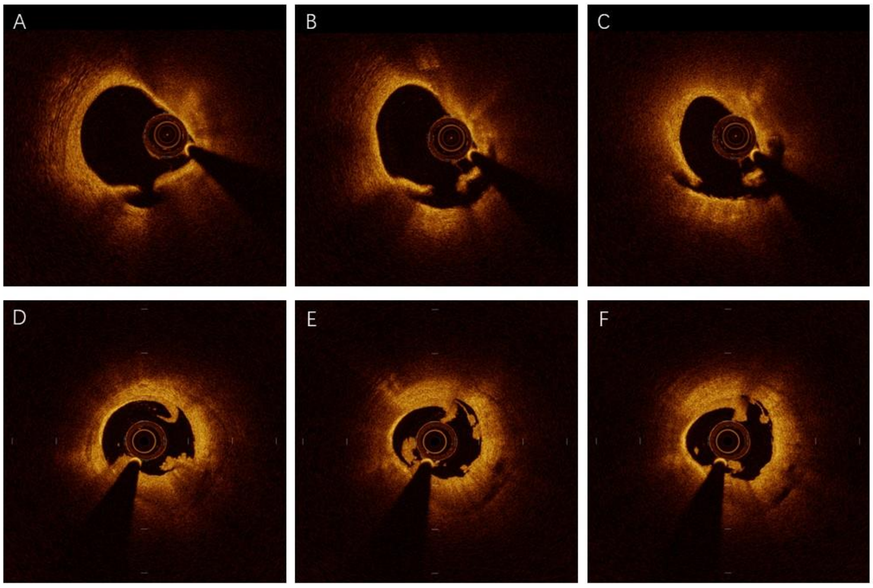

Supplemental Figure S1. Representative optical coherence tomography images for plaque rupture and non-plaque rupture. (A-C) Plaque rupture; (D-F) Non-plaque rupture.

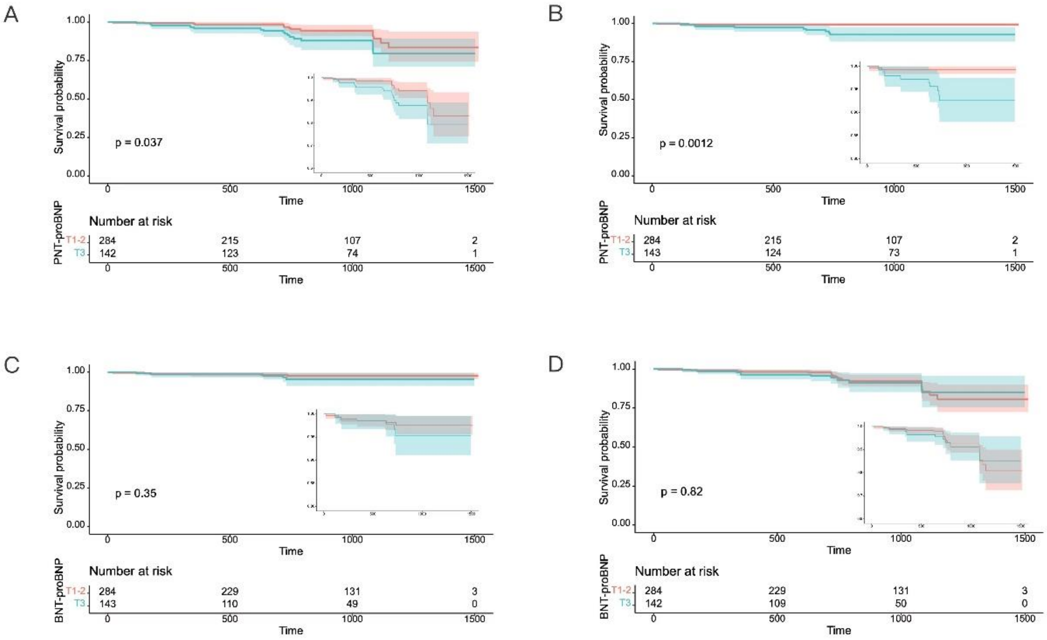

Supplemental Figure S2. Kaplan-Meier curves for cumulative rates of MACE and death according to peak and baseline value of NT-proBNP. (A) MACE for peak value of NT-proBNP; (B) Death for peak value of NT-proBNP; (C) Death for baseline value of NT-proBNP; (D) MACE for baseline value of NT-proBNP. PNT-proBNP, peak value of NT-proBNP; BNT-proBNP, baseline value of NT-proBNP.

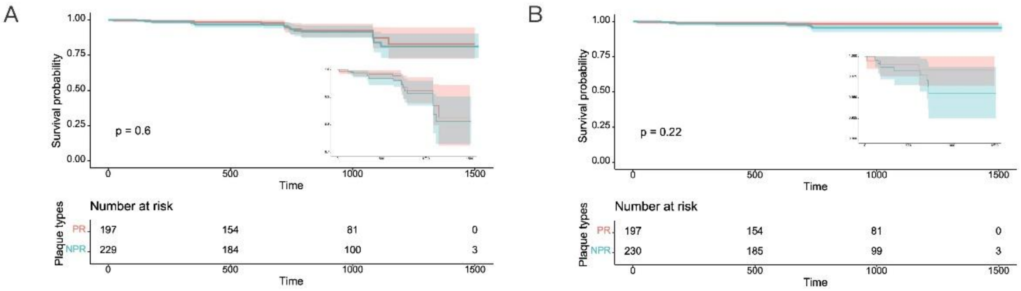

Supplemental Figure S3. Kaplan-Meier curves for cumulative rates of MACE and death according to plaque phenotype. (A) MACE for plaque rupture; (B) Death for non-plaque rupture. PR, plaque rupture; NPR, non-plaque rupture.

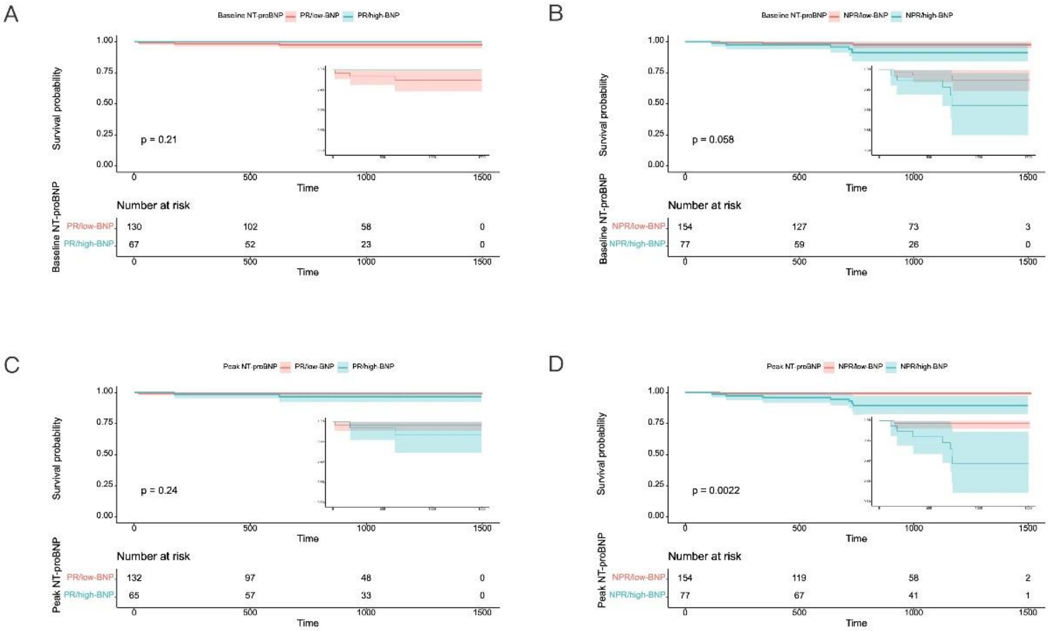

Supplemental Figure S4. Kaplan-Meier curves for cumulative death rate according to peak or baseline value of NT-proBNP and plaque phenotype. (A) Death for baseline value of NT-proBNP in patients with PR; (B) Death for baseline value of NT-proBNP in patients with NPR; (C) Death for peak value of NT-proBNP in patients with PR; (D) Death for peak value of NT-proBNP in patients with NPR. PR, plaque rupture; NPR, non-plaque rupture.

Supplemental Table S1. Hazard Ratio to MACE According to peak value of NT-proBNP in Plaque Rupture and Non-Plaque rupture

Group	Crude model		Adjust model I		Adjust model II		Adjust model III	
	Crude HR (95%CI)	Crude p value	Adjust I HR (95%CI)	Adjust p value	Adjust II HR 95%CI)	Adjust p value	Adjust III HR (95%CI)	Adjust p value
**PR[#]**								
Death	3.824(0.346-42.243)	0.274	2.739(0.241-31.122)	0.416	1.831(0.131-25.632)	0.653	1.423(0.033-62.052)	0.855
MI	1.476(0.330-6.600)	0.610	1.100(0.240-5.030)	0.902	1.040(0.178-6.056)	0.966	1.343(0.103-17.501)	0.822
Stroke	0.551(0.057-5.307)	0.606	0.430(0.043-4.276)	0.472	0.321(0.027-3.796)	0.367	1.397(0.040-49.058)	0.854
MACE composite	1.366(0.473-3.943)	0.564	0.965(0.325-2.863)	0.949	0.976(0.322-2.961)	0.966	0.788(0.219-2.837)	0.716

**NPR#**								
Death	12.688(1.558-103.324)	0.018*	9.319(1.104-78.673)	0.040*	11.789(1.318-105.473)	0.027*	15.943(1.220-208.347)	0.035*
MI	1.437(0.320-6.440)	0.636	1.292(0.275-6.077)	0.746	1.889(0.344-10.379)	0.464	2.253(0.153-33.085)	0.554
Stroke	2.385(0.532-10.692)	0.256	2.287(0.484-10.800)	0.296	2.050(0.427-9.846)	0.370	2.238(0.399-12.548)	0.360
MACE composite	2.629(1.086-6.363)	0.032*	2.372(0.943-5.966)	0.066	2.793(1.059-7.367)	0.038*	3.328(1.019-10.868)	0.046*

Data presented are HRs and 95% CI. Adjust I model adjusts for sex and age; Adjust II model adjusts for adjust I plus smoke, hypertension, hyperlipidemia, diabetes mellitus and BMI; Adjust III model adjusts for adjust II + white blood cell counts, creatine kinase, glycosylated hemoglobin, high sensitivity C-reactive protein and left ventricle ejection fraction.

#Tertlie 3 NT-proBNP vs. Tertile 1-2 NT-proBNP

PR indicated plaque rupture; NPR indicated no plaque rupture

Supplemental Table S2. Discrimination and Reclassification of 2-Year death by Different Models

Models	NRI (95%CI)	p value	IDI (95%CI)	p value
Risk factors	Ref.	-	Ref.	-
Risk factors+NT-proBNP	-0.002(-0.011-0.006)	0.564	0.003(-0.006-0.0013)	0.512
Risk factors+NT-proBNP+plaque type	0.268(0.004-0.531)	0.046	0.051(0.010-0.092)	0.016

Notes: Risk factors included age, gender, hypertension, hyperlipidemia, diabetes, smoking, LDL, HsCRP, LVEF;

Abbreviations: MACE, major adverse cardiovascular events; NRI, net reclassification index; IDI, integrated discrimination improvement; CI, confidence interval.
